# Association of baseline and changes in health-related quality of life with mortality following myocardial infarction: multicentre longitudinal linked cohort study

**DOI:** 10.1093/ehjqcco/qcae036

**Published:** 2024-08-30

**Authors:** Tatendashe B Dondo, Theresa Munyombwe, Ben Hurdus, Suleman Aktaa, Marlous Hall, Anzhela Soloveva, Ramesh Nadarajah, Mohammad Haris, Robert M West, Alistair S Hall, Chris P Gale

**Affiliations:** Leeds Institute of Cardiovascular and Metabolic Medicine, University of Leeds, Leeds LS2 9JT, UK; Leeds Institute for Data Analytics, University of Leeds, Leeds LS2 9JT, UK; Leeds Institute of Cardiovascular and Metabolic Medicine, University of Leeds, Leeds LS2 9JT, UK; Leeds Institute for Data Analytics, University of Leeds, Leeds LS2 9JT, UK; Leeds Institute of Cardiovascular and Metabolic Medicine, University of Leeds, Leeds LS2 9JT, UK; Leeds Institute for Data Analytics, University of Leeds, Leeds LS2 9JT, UK; Leeds Institute of Cardiovascular and Metabolic Medicine, University of Leeds, Leeds LS2 9JT, UK; Department of Cardiology, Leeds General Infirmary, Leeds LS1 3EX, UK; Leeds Institute of Cardiovascular and Metabolic Medicine, University of Leeds, Leeds LS2 9JT, UK; Leeds Institute for Data Analytics, University of Leeds, Leeds LS2 9JT, UK; Leeds Institute of Cardiovascular and Metabolic Medicine, University of Leeds, Leeds LS2 9JT, UK; Leeds Institute for Data Analytics, University of Leeds, Leeds LS2 9JT, UK; Leeds Institute of Cardiovascular and Metabolic Medicine, University of Leeds, Leeds LS2 9JT, UK; Leeds Institute for Data Analytics, University of Leeds, Leeds LS2 9JT, UK; Department of Cardiology, Leeds General Infirmary, Leeds LS1 3EX, UK; Leeds Institute of Cardiovascular and Metabolic Medicine, University of Leeds, Leeds LS2 9JT, UK; Leeds Institute for Data Analytics, University of Leeds, Leeds LS2 9JT, UK; Leeds Institute of Health Sciences, University of Leeds, Leeds LS2 9JT, UK; Leeds Institute of Cardiovascular and Metabolic Medicine, University of Leeds, Leeds LS2 9JT, UK; Leeds Institute of Cardiovascular and Metabolic Medicine, University of Leeds, Leeds LS2 9JT, UK; Leeds Institute for Data Analytics, University of Leeds, Leeds LS2 9JT, UK; Department of Cardiology, Leeds General Infirmary, Leeds LS1 3EX, UK

**Keywords:** Myocardial infarction, Health related quality of life, EQ-5D, Mortality, Prognosis, EMMACE, MINAP

## Abstract

**Background:**

Health-related quality of life (HRQoL) for patients following myocardial infarction (MI) is frequently impaired. We investigated the association of baseline and changes in HRQoL with mortality following MI.

**Methods and results:**

Nationwide longitudinal study of 9474 patients admitted to 77 hospitals in England as part of the Evaluation of the Methods and Management of Acute Coronary Events study. Self-reported HRQoL was collected using EuroQol EQ-5D-3L during hospitalization and at 1, 6, and 12 months following discharge. The data was analysed using flexible parametric and multilevel survival models. Of 9474 individuals with MI, 2360 (25%) were women and 2135 (22.5%) died during the 9-year follow-up period. HRQoL improved over 12 months (baseline mean, mean increase: EQ-5D 0.76, 0.003 per month; EQ-VAS 69.0, 0.5 per month). At baseline, better HRQoL was inversely associated with mortality [Hazard ratio (HR) 0.55, 95% CI 0.47–0.63], and problems with self-care (HR 1.73, 1.56–1.92), mobility (1.65, 1.50–1.81), usual activities (1.34, 1.23–1.47), and pain/discomfort (1.34, 1.22–1.46) were associated with increased mortality. Deterioration in mobility, pain/discomfort, usual activities, and self-care over 12 months were associated with increased mortality (HR 1.43, 95% CI 1.31–1.58; 1.21, 1.11–1.32; 1.20, 1.10–1.32; 1.44, 1.30–1.59, respectively).

**Conclusion:**

After MI, poor HRQoL at baseline, its dimensions, and deterioration over time are associated with an increased risk of mortality. Measuring HRQoL in routine clinical practice after MI could identify at-risk groups for interventions to improve prognosis.

## Graphical Abstract

**Graphical Abstract:**
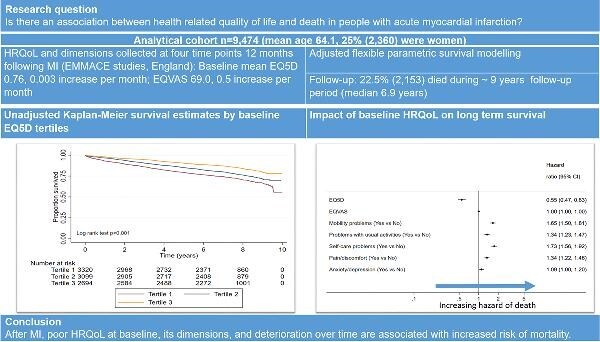

Key learning points What is already known

- Following myocardial infarction (MI), health-related quality of life (HRQoL) is frequently impaired and often deteriorates.
- There is limited health system-wide information about the association of HRQoL and mortality after MI.

What this study adds

- This prospective longitudinal linked data shows the negative impact of poor baseline HRQoL in each of the dimensions of EQ-5D on survival and how deterioration in these dimensions is associated with a worse prognosis.
- The systematic measurement of HRQoL following MI may offer actionable insights for patient stratification and predicting outcomes.

## Introduction

Quality of life predicts survival and rehospitalization for a range of major health conditions including cancer,^1^ pulmonary disease,^2^ renal disease,^3^ and organ transplantation.^4,5^ Herein, its routine measurement in clinical practice has been used to monitor disease progression, identify patients at risk of adverse outcomes, and highlight unforeseen problems due to prescribed medications,^6^ thus enabling stratified care with subsequent improvements in clinical outcomes.^1,7–9^ Yet, evidence is lacking for the association of health-related quality of life (HRQoL) with the prognosis for individuals admitted to the hospital with myocardial infarction (MI). This is important because early death following hospitalized MI has declined,^10,11^ resulting in a much later and higher burden of mortality, morbidity, and healthcare utilization.^12^ Given that MI remains a common reason for hospitalization,^12,13^ new strategies to improve latent health outcomes in this group are required.

Following MI, HRQoL is frequently impaired and often deteriorates.^14^ This includes greater physical limitation and more problems with self-care, pain, and mental stress.^14,15^ Poor HRQoL can persist after an initial cardiac event^14,15^ and is associated with high health resource utilization.^16^ Moreover, specific baseline patient characteristics are associated with HRQoL trajectories following MI, which form unique recovery patterns. Our earlier research found that women, those with non-ST-elevation myocardial infarction (NSTEMI), and those with long-term health conditions were less likely to show improvements in HRQoL, and that distinct multimorbidity clusters were associated with HRQoL.^14,17^ To date, however, the association between HRQoL and clinical events in patients with MI is unknown. Furthermore, the absence of systematic capture of HRQoL for individuals with MI not only precludes novel observational insights into its relationship with prognosis, but hinders opportunities to integrate data-driven strategies based on patient perspectives to transform health provision.

The Evaluation of the Methods and Management of Acute Coronary Events, EMMACE-3 and EMMACE-4 cohorts are multicentre longitudinal studies of outcomes following MI that combine survey data with routine national health data and include information about HRQoL.^18^ There are few large-scale datasets available that combine clinical data with a robust evaluation of temporal changes in HRQoL for patients with MI. We therefore used the EMMACE cohorts to extended previous research to investigate associations between HRQoL and mortality in patients hospitalized with MI.

## Methods

### Setting and design

The study was based on the analysis of data from 9474 patients who participated in the EMMACE-3 and 4 nationwide longitudinal cohort studies. Details of the study have been published previously.^18^ In brief, all adults aged ≥18 years admitted with ST-elevation myocardial infarction (STEMI) or NSTEMI to 77 National Health Service (NHS) hospitals in England between 1 November 2011 and 24 June 2015 were eligible to be included. The study collected data relating to medication adherence, HRQoL, co-morbidities, treatments, and clinical outcomes for patients at hospitalization and then at 1-, 6-, and 12 months following hospital discharge. Records for consenting patients were linked to the United Kingdom (UK) national heart attack register (Myocardial Ischaemia National Audit Project, MINAP^19^) to gather data about past medical history, type of MI, and in-hospital treatment. All-cause mortality data, with a censoring date of when the research coordinator did the mortality tracking for each participant, was collected using the NHS Spine platform and linked to the EMMACE-3 and 4 data used for this study.

### Assessment of health-related quality of life

Self-reported HRQoL was quantified using EQ-5D-3L, a standardized instrument developed by the EuroQoL group and validated in post-MI patients.^20,21^ EQ-5D-3L is a descriptive classification made up of five dimensions: mobility, self-care, usual activities, pain/discomfort, and anxiety/depression. Each dimension is divided into three levels (3L): no problems, some problems, and extreme problems, indicating the patient's perceived level of function.^20^ The EQ-5D index score ranges from −0.5 to 1, with scores less than 0 indicating states ‘worse than death’, 0 indicating no quality of life or ‘death’ and 1 indicating full health, and therefore no problems in any domain. EQ-VAS is an analogue scale of 0–100 in which participants are required to indicate their own perceived health with 0 indicating ‘worst imaginable health state’ and 100 ‘best imaginable health state’.^20^

### Statistical analyses

Baseline characteristics were described using frequencies and proportions for categorical data. Normally distributed continuous data were described using means and standard deviations (SD), and non-normally distributed data using medians and interquartile ranges (IQR). For descriptive analyses, baseline HRQoL scores were categorized into tertiles. Latent growth models^22^ were used to describe changes in HRQoL over 12 months following MI, applying the lavaan package in R.

Flexible parametric^23^ and multilevel survival models were fitted to investigate the associations of baseline and change in HRQoL with survival. Adjustment in the models was made for: diabetes mellitus, hypercholesterolaemia, hypertension, asthma/chronic obstructive pulmonary disease (COPD), cerebrovascular disease, peripheral vascular disease, smoking status, family history of coronary heart disease (CHD), age, sex, care by cardiologist, previous percutaneous coronary intervention, previous coronary artery bypass graft surgery, previous myocardial infarction (MI), previous angina, chronic renal failure, chronic heart failure, and discharge medications (statins, aspirin, P2Y_12_ inhibitors, angiotensin converting enzyme inhibitors/angiotensin receptor blockers). To investigate the association between HRQoL domains and survival, domain responses were treated as binary variables, ‘some problems’ and ‘extreme problems’ categories vs. ‘no problems’. The scale (proportional hazards, proportional odds, or normal) and complexity (number of degrees of freedom) for flexible parametric survival models were checked on the full multivariable model. The baseline hazard on the hazard scale with five degrees of freedom produced the optimal model through minimization of the AIC and BIC (Supplementary material online, *Table S1*). Models were fit for the primary outcome of all-cause mortality during the follow up period and for secondary outcomes: 1 month, 6 months, and 1 year all-cause mortality.

Multiple imputations by chained equations were used to handle missing data in variables such as age and sex. Missing data in select binary treatment and medical history variables were imputed to ‘no’. Details of the imputation strategy applied to handle missing data are provided in Supplementary material online, *Table S2*. Rubin's rules were used to pool the results estimates of 10 number of imputations and generate 95% confidence intervals. Analyses were performed using Stata MP64 version 17 (StataCorp, www.stata.com), R version 3.1.2, and R version 4.1.0. *P*-values <0.05 were considered statistically significant.

### Ethics

EMMACE-3 was given a favourable ethical opinion by the Leeds (Central) Research Ethics Committee (REC reference: 10/H1313/74), is registered on ClinicalTrials.gov (NCT0180827), and was adopted onto the National Institute for Health Research Comprehensive Research Network portfolio (9102). EMMACE-4 was given favourable ethical opinion by the West Midlands—Black Country Research Ethics Committee (REC reference: 12/WM/0431), is registered on ClinicalTrials.gov (NCT01819103), and was adopted onto the National Institute for Health Research Comprehensive Research Network portfolio (9102). All patients included in the study have provided consent to participate and for their data to be used for research by initialling consent statements on the front of the questionnaires used for data collection.

### Patient and public involvement

The Leeds Teaching Hospitals NHS Trust Cardiovascular Patient and Public Involvement group was involved in the project design including the setting up of the EMMACE studies. Scheduled discussions were held with the group about the study and its potential impact. Feedback was received on how to best conduct the study to ensure patient benefit.

## Results

### Participant characteristics

Of the 9474 participants, the mean age was 64.1 (SD 12.0) years, 2360 (25.0%) were women, and 3875 (40.9%) had STEMI. Overall, the comorbidity burden of the cohort was high, and many had long-term health conditions. Two thirds of patients were current or ex-smokers (6181, 67.1%), almost half had hypertension (4029, 44.8%), and almost a third had hypercholesterolaemia (2911, 31.9%) (*Table 1*). Compared with participants in the highest tertile of HRQoL [EQVAS (>75) and EQ-5D (=1.0)] (good HRQoL) at baseline, those in the lowest tertile [EQVAS (≤55) and EQ-5D (≤0.69)] (poor HRQoL) less frequently had a STEMI, but more frequently were women and had higher rates of cardiovascular comorbidity and asthma/COPD (*Table 1*).

**Table 1:** Characteristics of study participants overall and, by baseline EQVAS and EQ-5D score tertiles

		EQVAS			EQ-5D			
Variable	Total cohort *N* = 9474	Tertile 1 (≤55) *n* = 3200	Tertile 2 (>55 to ≤75) *n* = 3276	Tertile 3 (>75) *n* = 2767	Tertile 1 (≤0.69) *n* = 3320	Tertile 2 (>0.69 to ≤0.88) *n* = 3099	Tertile 3 (=1.00) *n* = 2694	Missing *n* (%)
Age, mean (SD), year	64.1 (12.0)	63.6 (12.3)	64.7 (11.9)	64.0 (11.6)	64.3 (12.4)	65.0 (12.1)	62.7 (11.2)	17 (0.2)
Female, *n* (%)	2360 (25.0)	983 (30.8)	775 (23.7)	542 (19.6)	1045 (31.6)	761 (24.6)	463 (17.2)	23 (0.2)
Ex/current smoking status, *n* (%)	6181 (67.1)	2109 (67.9)	2122 (66.6)	1796 (66.8)	2225 (69.1)	1968 (65.2)	1748 (66.7)	261 (2.8)
Family history of CHD, *n* (%)	3118 (38.9)	1004 (38.0)	1072 (38.5)	956 (40.3)	989 (36.6)	1005 (38.4)	1005 (42.2)	1464 (15.5)
Comorbidities								
Previous PCI, *n* (%)	894 (10.0)	335 (11.2)	326 (10.5)	220 (8.3)	351 (11.4)	315 (10.7)	202 (7.8)	508 (5.4)
Previous CABG surgery, *n* (%)	642 (7.2)	246 (8.2)	223 (7.2)	163 (6.2)	266 (8.6)	221 (7.5)	134 (5.2)	494 (5.2)
Previous MI, *n* (%)	1512 (16.8)	562 (18.7)	521 (16.7)	390 (14.8)	602 (19.4)	505 (17.2)	344 (13.2)	484 (5.1)
Previous angina, *n* (%)	1778 (19.8)	702 (23.3)	603 (19.4)	432 (16.4)	741 (23.9)	602 (20.5)	366 (14.1)	491 (5.2)
Chronic renal failure, *n* (%)	286 (3.2)	113 (3.8)	97 (3.1)	67 (2.5)	130 (4.2)	87 (3.0)	53 (2.0)	497 (5.3)
Hypertension, *n* (%)	4029 (44.8)	1418 (47.1)	1386 (44.6)	1119 (42.4)	1486 (47.9)	1339 (45.5)	1053 (40.5)	485 (5.1)
Chronic heart failure, *n* (%)	211 (2.4)	91 (3.0)	68 (2.2)	45 (1.7)	116 (3.8)	58 (2.0)	27 (1.0)	500 (5.3)
Hypercholesterolemia, *n* (%)	2911 (31.9)	1012 (33.2)	1012 (32.0)	814 (30.3)	1050 (33.3)	958 (32.2)	796 (30.2)	351 (3.7)
Peripheral vascular disease, *n* (%)	314 (3.6)	130 (4.4)	97 (3.2)	81 (3.1)	148 (4.8)	104 (3.6)	52 (2.0)	622 (6.6)
Asthma/COPD, *n* (%)	1154 (12.9)	463 (15.5)	396 (12.7)	261 (9.9)	485 (15.7)	382 (13.0)	237 (9.1)	504 (5.3)
Cerebrovascular disease, *n* (%)	426 (4.7)	160 (5.3)	146 (4.7)	108 (4.1)	177 (5.7)	143 (4.9)	89 (3.4)	494 (5.2)
Diabetes mellitus, *n* (%)	1699 (18.6)	642 (20.8)	579 (18.2)	432 (16.2)	731 (22.9)	545 (18.2)	342 (13.2)	326 (3.4)
Final diagnosis (STEMI vs. NSTEMI)	3875 (40.9)	1261 (39.4)	1358 (41.5)	1176 (42.5)	1260 (38.0)	1251 (40.4)	1233 (45.8)	0 (0)
Treatments^a^								
Coronary intervention (PCI/CABG), *n* (%)	4284 (59.5)	1445 (59.9)	1444 (58.0)	1287 (60.5)	1385 (57.2)	1420 (60.8)	1316 (60.3)	1570 (17.9)
Discharge medications^a^								
Aspirin, *n* (%)	8071 (99.3)	2647 (99.2)	2814 (99.3)	2415 (99.5)	2744 (99.0)	2611 (99.4)	2405 (99.6)	302 (3.6)
Beta blocker, *n* (%)	7524 (98.3)	2457 (98.1)	2621 (98.4)	2271 (98.6)	2536 (98.1)	2441 (98.3)	2266 (98.7)	315 (4.0)
Statins, *n* (%)	8065 (99.1)	2650 (99.0)	2805 (99.2)	2414 (99.1)	2756 (99.0)	2606 (99.0)	2396 (99.4)	311 (3.7)
ACEI/ARBs, *n* (%)	7535 (97.6)	2461 (97.2)	2602 (97.8)	2297 (97.7)	2543 (97.2)	2427 (97.6)	2281 (97.9)	342 (4.2)
P2Y_12_ inhibitors, *n* (%)	4816 (97.3)	1574 (97.0)	1685 (97.5)	1403 (97.6)	1615 (96.8)	1559 (97.6)	1434 (97.9)	479 (8.8)
Cardiac rehabilitation, *n* (%)	8424 (97.7)	2806 (97.0)	2907 (97.9)	2508 (98.3)	2863 (97.0)	2781 (98.0)	2464 (98.3)	606 (6.6)
HRQoL at follow-up								
EQ-5D, median (IQR)								
Admission	0.8 (0.6–1.0)	0.6 (0.4–0.8)	0.8 (0.7–1.0)	0.9 (0.8–1.0)	0.5 (0.2–0.6)	0.8 (0.8–0.8)	1.0 (1.0–1.0)	361 (3.8)
30 days	0.8 (0.6–1.0)	0.7 (0.6–0.8)	0.8 (0.7–1.0)	0.8 (0.7–1.0)	0.7 (0.5–0.8)	0.8 (0.7–0.9)	0.9 (0.8–1.0)	2979 (31.4)
6 months	0.8 (0.7–1.0)	0.8 (0.6–1.0)	0.8 (0.7–1.0)	1.0 (0.7–1.0)	0.7 (0.5–0.9)	0.8 (0.7–1.0)	1.0 (0.8–1.0)	4031 (42.6)
12 months	0.9 (0.7–1.0)	0.8 (0.6–1.0)	0.8 (0.7–1.0)	1.0 (0.7–1.0)	0.7 (0.5–1.0)	0.8 (0.7–1.0)	1.0 (0.8–1.0)	4557 (48.1)
EQVAS								
Admission	69.0 (50.0–80.0)	44.0 (30.0–50.0)	70.0 (60.0–70.0)	85.0 (80.0–90.0)	50.0 (40.0–70.0)	70.0 (50.0–80.0)	75.0 (65.0–86.0)	299 (3.2)
30 days	70.0 (60.0–80.0)	60.0 (50.0–75.0)	70.0 (60.0–80.0)	80.0 (70.0–90.0)	65.0 (50.0–75.0)	70.0 (60.0–80.0)	80.0 (70.0–90.0)	2958 (31.2)
6 months	78.0 (62.0–86.0)	70.0 (50.0–80.0)	75.0 (65.0–85.0)	83.0 (75.0–90.0)	70.0 (50.0–80.0)	80.0 (65.0–85.0)	80.0 (70.0–90.0)	4040 (42.6)
12 months	80.0 (65.0–90.0)	70.0 (50.0–80.0)	80.0 (69.0–86.0)	85.0 (75.0–90.0)	70.0 (50.0–80.0)	80.0 (68.0–87.0)	83.0 (75.0–90.0)	4571 (48.3)

CHD, coronary heart disease; PCI, percutaneous coronary intervention; CABG, coronary artery bypass graft; MI, myocardial Infarction; COPD, chronic obstructive pulmonary disease; STEMI, ST-elevation myocardial infarction; NSTEMI, non ST-elevation myocardial infarction; HRQoL, health related quality of life, ACEI/ARBs, angiotensin converting enzyme inhibitors/angiotensin receptor blockers.

a Of the eligible.

### HRQoL at baseline and trajectories following MI

The median EQ-5D score at baseline was 0.81 (IQR 0.59–1.00) and the median EQ-VAS score at baseline was 70.0 (IQR 50.0–80.0). There was an improvement in HRQoL over 12 months following MI (baseline mean, mean increase: EQ-5D 0.76, 0.003 per month; EQ-VAS 69.0, 0.5 per month) (Supplementary material online, *Figure S1*). Stratified by sex, the baseline EQ-5D score mean for men was 0.74 (SD 0.28) and for women 0.66 (SD 0.31). By comparison, the mean EQ-5D score for an age-matched UK general population was 0.88 for men and 0.86 for women. At 12-month follow-up, the mean EQ-5D scores remained below the UK age and sex matched general population mean (Supplementary material online, *Figure S2*).

### Changes in HRQoL by tertile

**Figure 1:**
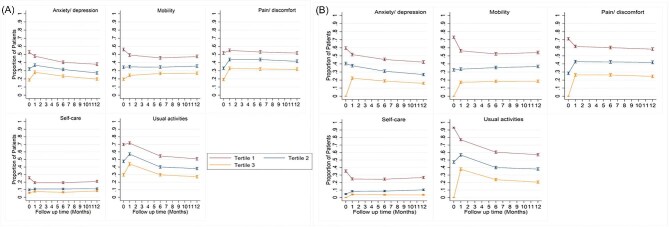
Health related quality of life domains trajectories following myocardial infarction by (*A*) EQ5D and (*B*) EQVAS tertiles.

Poor HRQoL persisted for patients in the lowest baseline tertile of HRQoL during follow-up (*Table 1*), and these patients were more likely to report problems in all dimensions of EQ-5D, with the highest frequencies observed for usual activities, mobility, pain/discomfort, and anxiety/depression (*Figure 1*). An increase in the frequencies of patients reporting problems with mobility, pain/discomfort, anxiety/depression, and usual activities was observed at 30 days for patients in the highest tertile of HRQoL at baseline (*Figure 1*).

### Mortality

**Figure 2:**
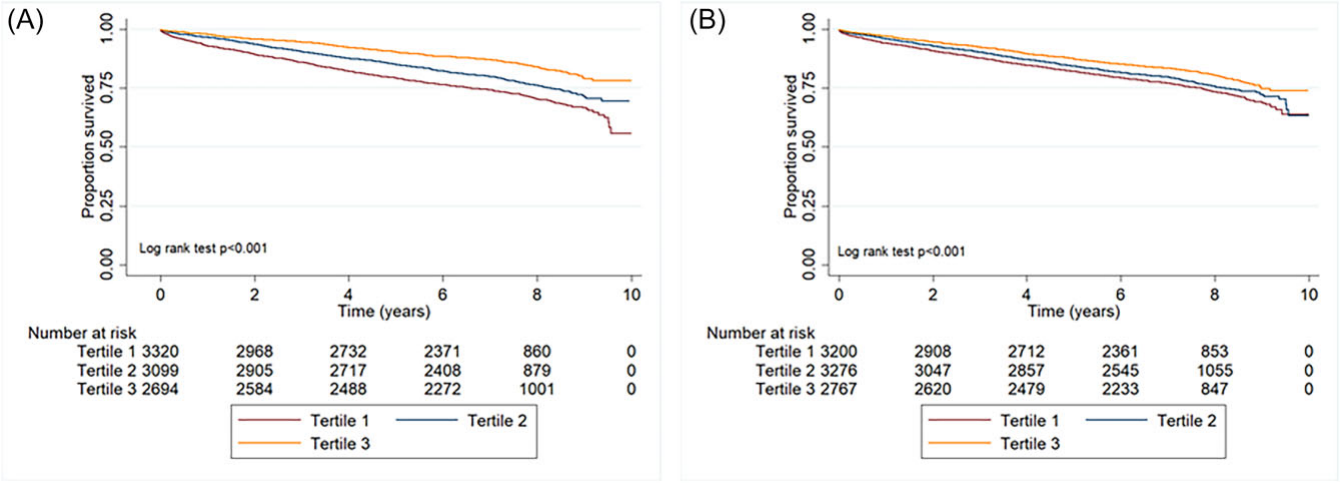
Unadjusted Kaplan-Meier survival estimates by HRQoL tertiles (*A*): by EQ5D tertiles and (*B*): by EQVAS tertiles.

Over 62 469 person-years, with a median duration of follow-up 6.9 (IQR 6.1–8.4) years, 2135 (22.5%) participants died. Mortality rates at 30 days, 6 months, and 12 months were 0.9% (81), 2.6% (245), and 4.2% (398), respectively. Compared with participants who were in the highest tertile of baseline HRQoL, those in the lowest tertile had higher unadjusted mortality rates (EQ-5D: 28.0 vs. 15.3% and EQ-VAS: 25.0 vs. 18.6%, *P* < 0.001) and demonstrated differences in unadjusted survival (*Figure 2*).

### Association of baseline HRQoL with mortality

**Figure 3:**
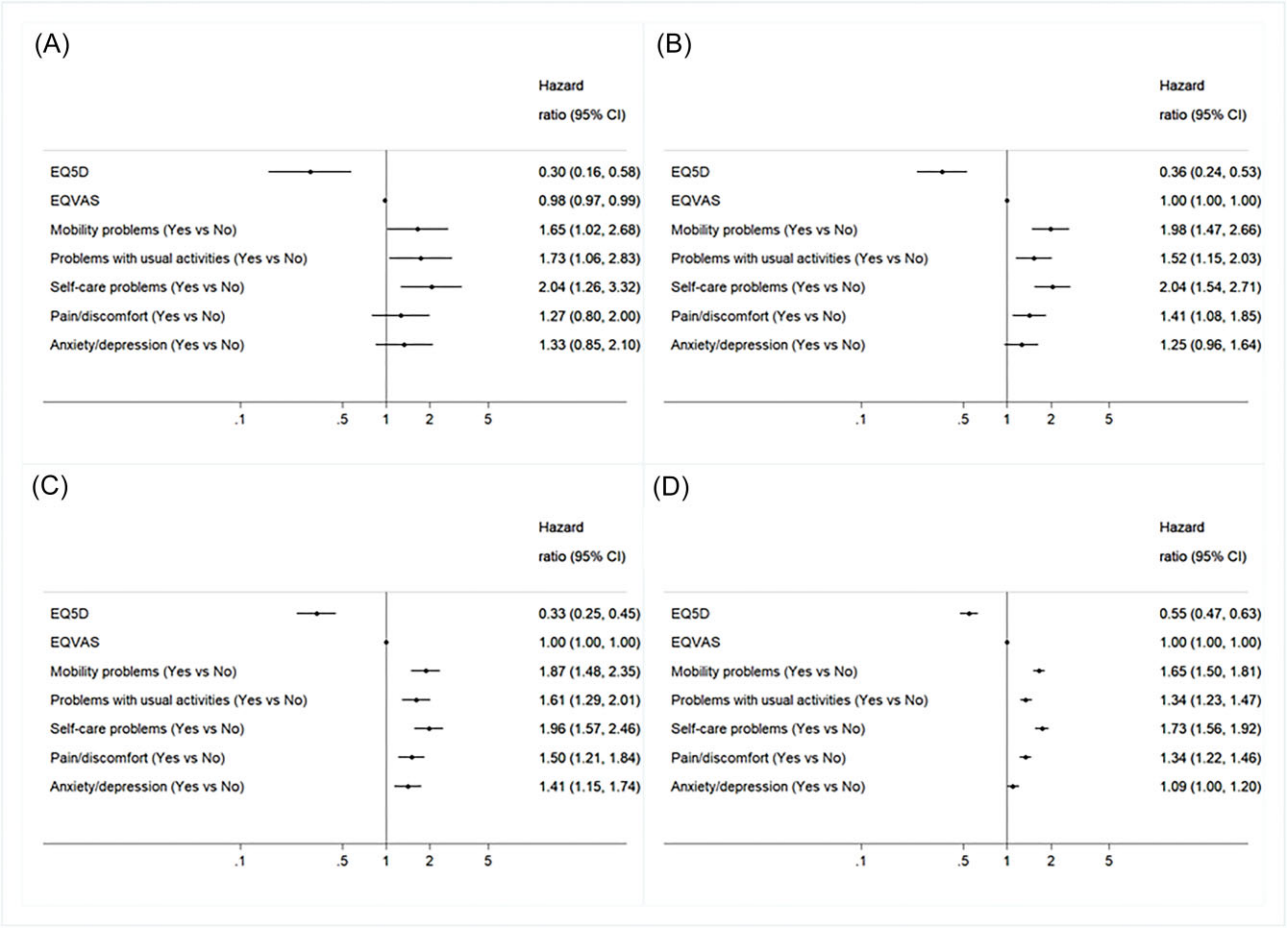
Impact of baseline HRQoL on survival (*A*) 30 day, (*B*) Six months, (*C*) 12 months, and (*D*) long term survival.

A 0.1 increase in baseline EQ-5D was associated with reduced mortality (adjusted HR 0.55, 95% CI 0.47–0.63) (*Figure 3*), but no statistically significant association was observed between baseline EQ-VAS score and survival. For 30-day survival, problems with mobility (HR 1.65, 1.02–2.68), usual activities (HR 1.73, 1.06–2.83), and self-care (HR 2.04, 1.26–3.32) were associated with an increased risk of death. Pain/discomfort were also associated with an increased risk of death at 6 months (HR 1.41, 1.08–1.85). Each of the dimensions of poor HRQoL was associated with an increased risk of death at 12 months and in the longer term (*Figure 3*), with the exception of anxiety/depression at baseline, which was associated with increased risk of death at 12 months alone (HR 1.41, 1.15–1.74).

### Association of changes in HRQoL with mortality

Improvements in HRQoL following MI were associated with improved survival. Overall, improvement in EQ-5D during 12-month follow-up was associated with a 31% reduction in risk of death (adjusted HR 0.69, 95% CI 0.60–0.80) (*Table 2*). No statistically significant association was observed for changes in EQ-VAS and survival. Patients with a deterioration in mobility, pain/discomfort, usual activities, and self-care during the 12 months of follow-up were at increased risk of death compared with those not reporting deterioration (HR 1.43, 95% CI 1.31–1.58; 1.21, 1.11–1.32; 1.20, 1.10–1.32; 1.44, 1.30–1.59; respectively) (*Table 2*).

**Table 2:** Impact of changes in HRQoL 12 months following MI on long term survival

	Hazard ratio (95% CI)	*P* value
Model		
EQ 5D	0.69 (0.60–0.80)	<0.001
EQ VAS	0.999 (0.999–1.000)	0.706
EQ 5D dimensions (yes vs. no)		
Mobility problems		
No	Ref	
Yes	1.43 (1.31–1.58)	<0.001
Problems with usual activities		
No	Ref	
Yes	1.20 (1.10–1.32)	<0.001
Self-care problems		
No	Ref	
Yes	1.44 (1.30–1.59)	<0.001
Pain/discomfort		
No	Ref	
Yes	1.21 (1.11–1.32)	<0.001
Anxiety/depression		
No	Ref	
Yes	1.03 (0.94–1.13)	0.494

## Discussion

### Principal findings

In this national longitudinal cohort study of 9474 patients admitted with MI to 77 hospitals in England, we found that higher HRQoL measured using EQ-5D, at baseline and over time was associated with better survival. Specific dimensions of HRQoL captured by EQ-5D including problems with mobility, usual activities, and self-care, have prognostic implications in the short- and long-term after MI. This study found that deterioration in mobility, usual activities, pain/discomfort, and self-care, but not mental health, were independently associated with adverse prognosis.

### Comparison with other studies

Poor HRQoL following MI is well described.^14,15^ An observational study of 8978 participants with MI found that almost half reported ‘some’ or ‘severe’ problems on at least one dimension of their health status.^15^ This impairment in HRQoL after MI persists over time in about a third of patients.^14^ Previous studies have shown that patient-reported health status measures are associated with clinical outcomes in patients with cardiovascular disease,^15,24–26^ but there is little health system-wide information about the association of HRQoL and mortality. One study did find that lower EQ-5D, but not EQVAS, was associated with a higher risk of death and a composite of major cardiovascular events over 2 years post-MI.^15^ Our findings extend this by demonstrating in a large prospective study the detrimental impact of poor baseline HRQoL in each of the dimensions of EQ-5D on short and longer term outcomes and how deterioration in these dimensions is associated with a worse prognosis.

Our study did not find an association between anxiety/depression and mortality. This is in keeping with previous reports in which self-reported anxiety and depression did not predict cardiovascular outcomes.^15,27^ The findings could be attributed to improvements in the management of anxiety/depression in the contemporary era, e.g. with the prescription of antidepressants, but are in contrast to the finding that hospitalization with neuro-psychiatric diagnoses (including anxiety and depression) following circulatory disorders are more common among patients with MI, and this is associated with increased mortality compared with non-MI matched controls.^12^

Compared to a UK age- and sex-matched general population, HRQoL was lower for MI patients at hospitalization. It improved during follow-up though remained lower than the age- and sex-matched general population average. The lower HRQoL observed in the patients could be due to impact of MI and higher comorbidity burden. In a previous study we conducted, we found that reduced HRQoL in MI patients was associated with chronic renal failure, COPD, cerebrovascular disease, previous angina, and previous MI,^17^ all of which are highly prevalent in survivors of MI.^17^

### Implications for practice

Evidence supporting the incorporation of health status in risk stratification for MI is lacking, and current efforts to systematically capture HRQoL after MI are at best minimal. Our study adds to the growing evidence that HRQoL after MI is an important variable that may be used in predicting clinical outcomes.^15,25,26,28,29^ There is divergence between patients’ and physicians’ perceptions of patients’ health status. As such, patient-reported outcome measures add a fundamental value to risk assessment and mitigation. A key finding of this study is that improvements in HRQoL are associated with favourable clinical outcomes, and that deterioration in measurable parameters of HRQoL are associated with adverse prognosis. Assessing HRQoL routinely could help identify patients with MI who are at higher risk of premature death, allowing targeted identification of individuals who may be suitable for intervention. For example, information about HRQoL could be used to encourage enrolment into tailored programmes of cardiac rehabilitation, identify those who may be at future risk of non-adherence to medications, or schedule more frequent clinical reviews. A prospective evaluation of interventions guided by the baseline and trajectory of HRQoL is required to determine the clinical and cost-effectiveness of such an approach.

Nonetheless, challenges exist in implementing HRQoL assessment following MI. First, the time and effort required to collect data pertinent to HRQoL may create a burden on already overstretched healthcare systems. The establishment of quality indicators for the assessment of HRQoL after MI by professional organizations,^30^ may promote healthcare regulators to develop strategies for the collection of this important aspect of MI care. Whilst the EQ-5D is a generic HRQoL questionnaire, it has been validated for MI patients and provides the ability to compare HRQoL impairment with other cardiovascular and non-cardiovascular diseases. Whilst our study has shown that the EQ-5D-3L score can predict the risk of all-cause mortality in patients with MI, and that individual dimensions provide important prognostic information, it could be improved upon by data collection that captures health status dimensions specific to MI survivors (e.g. burden of medication and angina).

### Strengths and limitations

Our study benefits from a nationwide longitudinal cohort with longer term follow-up in terms of outcomes than has been previously reported. The use of a nationwide dataset increases the generalizability of the results. However, we do acknowledge that there will have been bias in recruitment and this will have been reflected in the findings—only those who survive to hospitals discharge could participate, and case selection will have occurred resulting in a younger and healthier cohort of people with MI. Loss of follow-up data can also introduce selection bias, which may have affected the magnitude of associations we observed. The external validity of this study could be limited by the fact that data collection for HRQoL following MI information was conducted between November 2011 and June 2015. However, the outcome follow-up data was censored in 2020 which relates to contemporary practice.

## Conclusions

This nationwide prospective cohort study found that better and improved HRQoL following MI was associated with improved survival. Patients’ perspective of their own well-being is an important variable following MI, which can be incorporated into routine care to guide risk stratification, targeted identification, and tailored treatment strategies.

## Supplementary Material

qcae036_Supplemental_File

## Notes

**Conflict of interest:** I have read the journal's policy and the authors of this manuscript have the following competing interests: C.P.G. reports study funding from Horizon 2020. C.P.G. reports grants from Alan Turing Institute, British Heart Foundation, National Institute for Health Research, Horizon 2020, Abbott Diabetes, Bristol Myers Squibb, and European Society of Cardiology. C.P.G. reports consulting fees from AI Nexus, AstraZeneca, Amgen, Bayer, Bristol Myers Squibb, Boehrinher-Ingleheim, CardioMatics, Chiesi, Daiichi Sankyo, GPRI Research B.V., Menarini, Novartis, iRhythm, Organon, and The Phoenix Group. C.P.G. reports payment or honoraria for lectures, presentations, speakers bureaus, manuscript writing or educational events from AstraZeneca, Boston Scientific, Menarini, Novartis, Raisio Group, Wondr Medical, and Zydus. C.P.G. reports receiving support for attending meetings and/or travel from AstraZeneca. C.P.G. reports participation on a Data Safety Monitoring Board or Advisory Board for DANBLCOK trial and TARGET CTCA trial. C.P.G. reports leadership or fiduciary role in other board, society, committee or advocacy group, paid or unpaid as deputy editor: EHJ Quality of Care and Clinical Outcomes, NICE Indicator Advisory Committee and Chair ESC Quality Indicator Committee. C.P.G. reports stock or stock options with CardioMatics. C.P.G. reports receipt of equipment, materials, drugs, medical writing, gifts or other services from Kosmos device. M.H. reports grants from Wellcome Trust, Alan Turing Institute, and British Heart Foundation. T.M. reports grant from British Heart Foundation. S.A. reports support for attending meetings and/or travel from European Society of Cardiology. S.A. reports participation on a Data Safety Monitoring Board or Advisory Board for Data management and analysis group of British Cardiovascular Intervention Society. A.S. reports grants or contracts from European Society of Cardiology. A.S. reports payment or honoraria for; lectures from AstraZeneca and Gedeon Richter, for manuscript writing from Bayer and for educational events from Novartis. A.S. reports support for attending meetings and travel from Servier and Amgen. A.S. reports leadership or fiduciary role in other board, society, committee or advocacy group, paid or unpaid for AstraZeneca. The remaining authors have nothing to disclose. All authors have completed the ICMJE uniform disclosure form at www.icmje.org/coi_disclosure.pdf.

## Data availability

No additional data are available.
