# Supplementary material for: Association of baseline and changes in health-related quality of life with mortality following myocardial infarction: multicentre longitudinal linked cohort study
Source: Eur Heart J Qual Care Clin Outcomes. 2024 Aug 30;11(6):730–8. doi: 10.1093/ehjqcco/qcae036 (PMC12445645; doi:10.1093/ehjqcco/qcae036)

**Supplementary Material**

Association of baseline and changes in health-related quality of life with mortality following myocardial infarction: Multicentre longitudinal linked cohort study

TB Dondo (0000-0002-8337-8425), T Munyombwe, R Nadarajah, B Hurdus, S Aktaa, A Soloveva, M Haris, M Hall, RM West, AS Hall, CP Gale.

[Supplementary Table 1 (complete cases): Choice of scale and degrees of freedom for the flexible parametric survival model determined by minimisation of the Akaike’s Information Criterion and Bayes Information Criterion. 2](#_Toc150119503)

[Supplementary Table 2: Study imputation strategy 3](#_Toc150119504)

[Supplementary Figure 1: Health related quality of life trajectories over 12 months following myocardial infarction by EQ5D and EQVAS. 6](#_Toc150119505)

[Supplementary Figure 2: Health related quality of life trajectories over 12 months following myocardial infarction by EQ5D compared to the age sex matched UK general population. 7](#_Toc150119506)

# Supplementary Table 1 (complete cases): Choice of scale and degrees of freedom for the flexible parametric survival model determined by minimisation of the Akaike’s Information Criterion and Bayes Information Criterion.

| **df** | **Normal** |  | **Odds** |  | **Hazard** |  |
| --- | --- | --- | --- | --- | --- | --- |
|  | **AIC** | **BIC** | **AIC** | **BIC** | **AIC** | **BIC** |
| 1 | 9610.73 | 9777.11 | 9371.45 | 9537.83 | 9294.11 | 9460.49 |
| 2 | 9379.58 | 9551.33 | 9266.55 | 9438.29 | 9231.54 | 9403.29 |
| 3 | 9330.54 | 9507.66 | 9229.03 | 9406.14 | 9207.64 | 9384.75 |
| 4 | 9327.49 | 9509.97 | 9226.61 | 9409.09 | 9206.15 | 9388.63 |
| 5 | 9312.34 | 9500.19 | 9211.63 | 9399.48 | **9191.98** | **9379.82** |

**Abbreviations:** AIC- Akaike’s Information Criterion; BIC- Bayes Information Criterion; df-degrees of freedom.

# Supplementary Table 2: Study imputation strategy

| **Variable** | **Variable Type** | **Missing n (%)** | **Imputation Method** |
| --- | --- | --- | --- |
| Sex | Binary | 23 (0.2) | Logistic regression |
| Age | Continuous | 17 (0.2) | Predictive mean matching |
| Ex/current smoking status | Binary | 261 (2.8) | Predictor/ Auxiliary and Default imputed |
| Family history of CHD | Binary | 1,464 (15.5) | Predictor/ Auxiliary and Default imputed |
| Previous PCI | Binary | 508 (5.4) | Predictor/ Auxiliary and Default imputed |
| Previous CABG | Binary | 494 (5.2) | Predictor/ Auxiliary and Default imputed |
| Previous AMI | Binary | 484 (5.1) | Predictor/ Auxiliary and Default imputed |
| Previous Angina | Binary | 491 (5.2) | Predictor/ Auxiliary and Default imputed |
| Chronic Renal Failure | Binary | 497 (5.3) | Predictor/ Auxiliary and Default imputed |
| Hypertension | Binary | 485 (5.1) | Predictor/ Auxiliary and Default imputed |
| Chronic Heart Failure | Binary | 500 (5.3) | Predictor/ Auxiliary and Default imputed |
| Hypercholesterolaemia | Binary | 351 (3.7) | Predictor/ Auxiliary and Default imputed |
| Peripheral Vascular Disease | Binary | 622 (6.6) | Predictor/ Auxiliary and Default imputed |
| Cerebrovascular Disease | Binary | 494 (5.2) | Predictor/ Auxiliary and Default imputed |
| Asthma or COPD | Binary | 504 (5.3) | Predictor/ Auxiliary and Default imputed |
| Diabetes | Binary | 326 (3.4) | Predictor/ Auxiliary and Default imputed |
| Final diagnosis | Binary | 0 (0) | Predictor/ Auxiliary and Default imputed |
| Coronary intervention (PCI/CABG) | Binary | 1,570 (17.9) | Predictor/ Auxiliary and Default imputed |
| Aspirin at hospital discharge | Binary | 302 (3.6) | Predictor/ Auxiliary and Default imputed |
| Beta blocker at hospital discharge | Binary | 315 (4.0) | Predictor/ Auxiliary and Default imputed |
| Statins at hospital discharge | Binary | 311 (3.7) | Predictor/ Auxiliary and Default imputed |
| ACEI/ARBs at hospital discharge | Binary | 342 (4.2) | Predictor/ Auxiliary and Default imputed |
| P2Y_12_ inhibitors at hospital discharge | Binary | 479 (8.8) | Predictor/ Auxiliary and Default imputed |
| Cardiac rehabilitation | Binary | 606 (6.6) | Predictor/ Auxiliary and Default imputed |
| EQVAS |  |  |  |
| Admission | Continuous | 361 (3.8) | Predictor/ Auxiliary |
| 30 days | Continuous | 2,979 (31.4) | Predictor/ Auxiliary |
| 6 months | Continuous | 4,031 (42.6) | Predictor/ Auxiliary |
| 12 months | Continuous | 4,557 (48.1) | Predictor/ Auxiliary |
| EQ-5D |  |  |  |
| Admission | Continuous | 231 (2.4) | Predictor/ Auxiliary |
| 30 days | Continuous | 2,861 (30.2) | Predictor/ Auxiliary |
| 6 months | Continuous | 3,968 (41.9) | Predictor/ Auxiliary |
| 12 months | Continuous | 4,524 (47.8) | Predictor/ Auxiliary |
| Mobility problems |  |  |  |
| Admission | Binary | 232 (2.5) | Predictor/ Auxiliary |
| 30 days | Binary | 2,869 (30.3) | Predictor/ Auxiliary |
| 6 months | Binary | 3,972 (41.9) | Predictor/ Auxiliary |
| 12 months | Binary | 4,490 (47.4) | Predictor/ Auxiliary |
| Problems with usual activities |  |  |  |
| Admission | Binary | 297 (3.1) | Predictor/ Auxiliary |
| 30 days | Binary | 2,900 (30.6) | Predictor/ Auxiliary |
| 6 months | Binary | 3,975 (42.0) | Predictor/ Auxiliary |
| 12 months | Binary | 4,491 (47.4) | Predictor/ Auxiliary |
| Self-care problems |  |  |  |
| Admission | Binary | 247 (2.6) | Predictor/ Auxiliary |
| 30 days | Binary | 2,885 (30.5) | Predictor/ Auxiliary |
| 6 months | Binary | 3,976 (42.0) | Predictor/ Auxiliary |
| 12 months | Binary | 4,495 (47.5) | Predictor/ Auxiliary |
| Pain/discomfort |  |  |  |
| Admission | Binary | 248 (2.6) | Predictor/ Auxiliary |
| 30 days | Binary | 2,891 (30.5) | Predictor/ Auxiliary |
| 6 months | Binary | 3,975 (42.0) | Predictor/ Auxiliary |
| 12 months | Binary | 4,517 (47.7) | Predictor/ Auxiliary |
| Anxiety/depression |  |  |  |
| Admission | Binary | 249 (2.6) | Predictor/ Auxiliary |
| 30 days | Binary | 2,884 (30.4) | Predictor/ Auxiliary |
| 6 months | Binary | 3,975 (42.0) | Predictor/ Auxiliary |
| 12 months | Binary | 4,491 (47.4) | Predictor/ Auxiliary |
| Nelson-Aalen survival estimate | Continuous | 0 | Predictor/ Auxiliary |
| Censoring indicator | Binary | 0 | Predictor/ Auxiliary |

**Abbreviations**: CHD, Coronary heart disease; PCI, percutaneous coronary intervention; CABG, coronary artery bypass graft; AMI, Acute Myocardial Infarction; COPD, chronic obstructive pulmonary disease; STEMI, ST-elevation myocardial infarction; NSTEMI, non ST-elevation myocardial infarction; HRQoL, health related quality of life

# Supplementary Figure 1: Health related quality of life trajectories over 12 months following myocardial infarction by EQ5D and EQVAS.


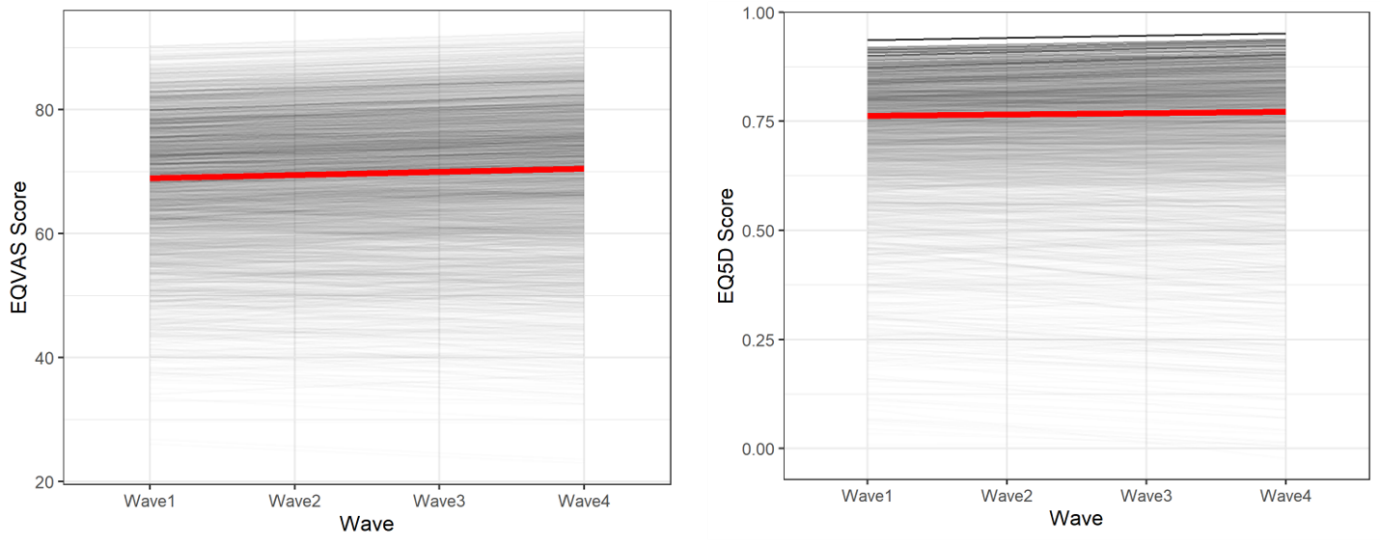


# Supplementary Figure 2: Health related quality of life trajectories over 12 months following myocardial infarction by EQ5D compared to the age sex matched UK general population.


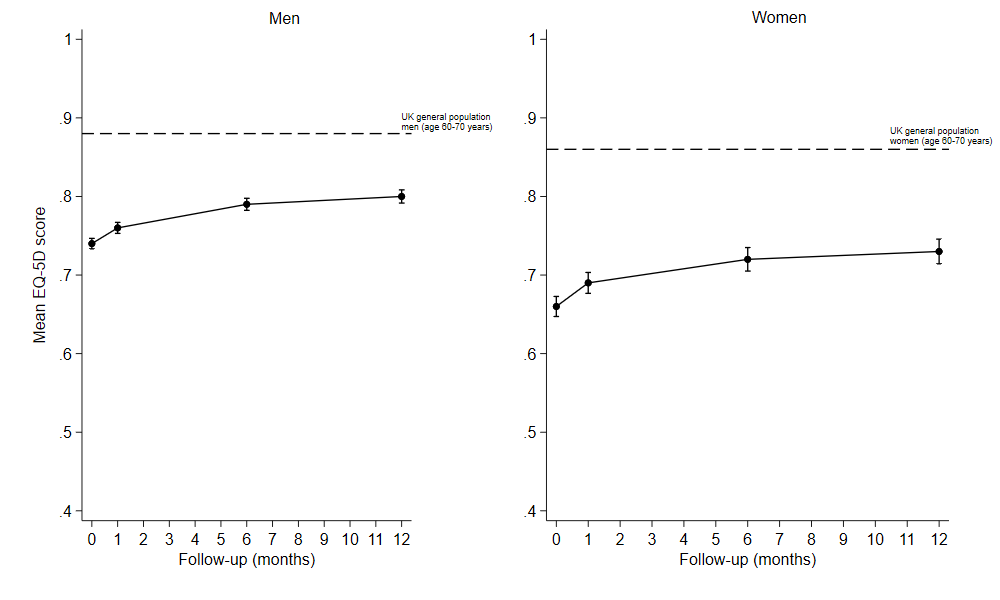

Supplement: qcae036_Supplemental_File [file qcae036_supplemental_file.docx]
